# Serious games for health promotion in adolescents – a systematic scoping review

**DOI:** 10.1007/s10639-022-11414-9

**Published:** 2022-11-04

**Authors:** Lesley Andrew, Donna Barwood, Julie Boston, Martin Masek, Lauren Bloomfield, Amanda Devine

**Affiliations:** 1 School of Nursing and Midwifery, Edith Cowan University, 270 Joondalup Dr, Joondalup, WA 6027 Australia; 2 School of Education, Edith Cowan University, Joondalup, Australia; 3 School of Science, Edith Cowan University, Joondalup, Australia; 4 School of Medical and Health Sciences, Edith Cowan University, Joondalup, Australia

**Keywords:** Health promotion, Adolescent, Serious games, Educational apps, Health education, Systematic scoping review

## Abstract

Digital gaming has broad appeal globally, with a reported 2.7 billion gamers worldwide. There is significant interest in using games to enhance learning, with ‘serious games’ being included in classrooms to engage adolescents’ learning across a range of domains. A systematic scoping review of serious games used for health promotion with adolescents was conducted to identify serious games, review the methods used to evaluate these games, and outline evidence available to support the efficacy of these games in improving knowledge, beliefs/attitudes and behaviours in the target groups. Player engagement/enjoyment was reported if assessed. A total of 21 studies were found to have met the inclusion criteria domains: ‘healthy lifestyle’ ‘sexual health’ and ‘substance use’. A heterogenous approach across studies to game design and development, duration of game play, use of a control group and measurement of outcome(s) was observed. Game efficacy was difficult to assess due to broad generalisations and lack of consistent evaluation methods. Several studies demonstrate serious games can be engaging and pedagogically effective as a learning device and behaviour-change agent. Several studies, however, had less rigorous evaluation and lacked longer-term follow up. The ability for developers to demonstrate positive short- and long-term impacts of serious games with high-quality evidence is essential to the ongoing acceptance and use of these serious games as part of the school curriculum.

## Introduction

Computer games have global appeal, with a reported 2.7 billion digital gamers worldwide (Deyan, 2021). They are particularly popular among adolescents, with 20% of people in the United States of America (USA) aged under 18 years (Statista, 2021), and > 67% of Australians aged 1–17 years reported to regularly play (Brand et al., 2019). There is significant interest in using games to enhance learning, with 52% of Australian children doing so as part of their classroom learning (Brand et al., 2019). Some commercial games have intrinsic educational value, where the same version distributed for entertainment can be used directly in the classroom. A large body of research exists to investigate how games can be designed specifically for a defined educational purpose. Such games fall under the broader umbrella term of ‘serious games’ (Abt, 1970), where the main purpose is education rather than entertainment. This does not mean serious games lack engaging and entertaining properties, but rather the addition of sound pedagogical practices sets them apart from games specifically designed to entertain (Zyda, 2005).

Serious games have an important role in health promotion, with games such as *Farm to Fork* (Edith Cowan University [ECU], 2020) (Australia) focusing on making healthier food choices and sustainable food production. Within the broader field of health promotion, complex and varied topics including sexual health, chronic disease, and mental health require specific and expert knowledge to facilitate and support cognition of health information and promote behaviour change. This ranges from understanding of strategies that can be used to explain difficult concepts, to pedagogical approaches that capture social and cultural nuances of communities and/or age groups.

The need to adapt complex health information for suitability with younger audiences, particularly adolescents, within an educational environment can pose further challenges for game developers. These include ways to maintain player engagement and ensuring the game reflects curricula and/or learning outcomes.

Supporting educators to deliver health promotion messages to adolescents using serious games acknowledges twenty-first century learning and popular culture, and potential of game play to tackle niche health issues. For example, *Farm to Fork* (ECU, 2020) specifically reflects learning outcomes articulated in the Australian Curriculum (Australian Curriculum, Assessment and Reporting Authority [ACARA], 2016) for Health and Physical Education (ACARA, 2015).

Designing serious games as pedagogical tools to support traditional learning and teaching modalities for health outcomes requires the evaluation of game efficacy to ensure it is on task, fit-for-purpose, effective as a resource for a range of schools and similar settings and is able to extend or complement learning.

Systematic reviews suggest many serious games for health promotion are not validated for their efficacy to improve health outcomes (DeSmet et al., 2015; Kato, 2012) and formal evaluation may not contain robust methodology limiting the generalisability of findings (Kato, 2012). These systematic reviews often focus on the use of serious games in specific domains within health promotion such as sexual health (DeSmet et al., 2015) or substance use (Rodriguez et al., 2014). These reviews have also included a wide range of delivery modalities outside of what would be considered ‘games’ and delivered across a wide range of age groups (DeSmet et al., 2014).

This scoping review focuses on the use of serious games aimed at an adolescent audience across a broad range of health promotion topics. Scoping reviews are a useful method of mapping a large disparate body of work that, because of its heterogeneity, is not amenable to the traditional systematic literature review that tends to focus on a narrow, defined topic (Arksey & O'Malley, 2005; Peters et al., 2020). This scoping review used a systematic approach that adheres to guidelines developed by Joanna Briggs Institute experts (Peters et al., 2015, 2020) to map serious games that target adolescent health promotion and assess their efficacy.

The scoping review addresses the following research questions:

1. What educational games could be identified in the literature that are used for health promotion with adolescents?
2. What methods were used to evaluate participant engagement and serious games efficacy?
3. What does the evidence show in terms of efficacy of these games in increasing knowledge/skills or changing behaviour/attitudes of adolescents?

## Materials and methods

The initial literature search and article selection process was undertaken by the research team using eligibility criteria and search terms. The protocol for this review has not been published elsewhere and is described below. Article retrieval, eligibility determination, and evaluation occurred from April 2020 to January 2022.

### Data repositories

Comprehensive searches of the ACM Digital Library, IEEE Xplore, ERIC, Medline and Education Source were performed. References of articles identified in the initial search, including systematic reviews and meta-analyses, were reviewed to determine if any further references met the eligibility criteria (Table 1) and suitable for inclusion.

**Table 1:** Eligibility criteria for inclusion in scoping review

Inclusion criteria	Exclusion criteria
Articles on serious games focused on health promotion	Articles about games or apps for rehabilitation, medical/science training and education, treatment for specific medical ailments, disabilities, illness, and articles investigating the impacts of excessive game playing
Peer-reviewed primary research articles discussing the development and evaluation of games	Online learning modules, computer assisted tools, web-based tools and other modalities not considered ‘games’
Articles published between 2005 and 2022	Articles not published in English
Conference proceedings were included	Opinion pieces, commentaries, and methods papers
Target/focus group of adolescents as defined by the World Health Organization and that is, persons aged between 10 and 19 years (Rodriguez et al., 2014)	Studies targeted primarily at ‘young adults’ who were post school-age were excluded
	Studies on ‘exergames’

### Search terms and strategy

1. The systematic scoping review employed a five-step search strategy (Arksey & O'Malley, 2005): To select an optimal set of search terms, a pre-review of the literature was conducted using the primary databases and/or repositories. This pre-review involved an iterative process, determining the initial set of search terms and informing suitability criteria.
2. These search terms were combined using a comprehensive set of expressions (terms and operators) commonly used in a systematic literature search. At this step, a search was performed of the title, abstract and full text to identify articles. The search expression subsequently used for the full review was as follows: “serious game*” OR “educational game*” OR “video game*” OR “educational app*” [in Abstract] AND health [in Abstract] AND adolescent* OR teen* OR “young person” OR youth OR student [in Abstract]
3. The returned articles were then scanned to confirm eligibility and inclusion.
4. The reference list of each article was then reviewed to identify additional articles for possible inclusion. The additional articles were reviewed to confirm inclusion.
5. To increase the likelihood that all relevant journal and peer-reviewed conference articles were captured, Google Scholar and Web of Science were searched using the same search terms and iterative process.

**Fig. 1:**
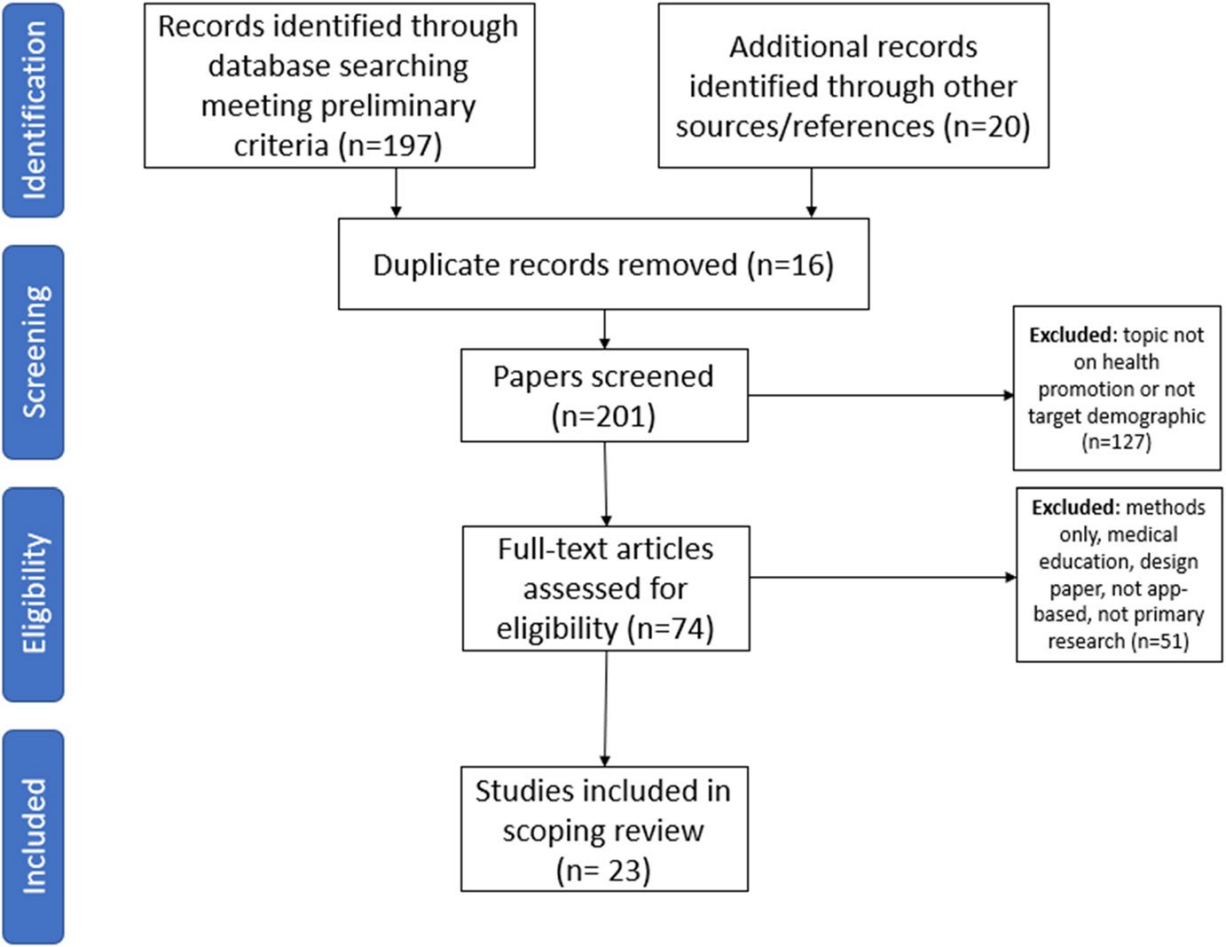
Flowchart of article retrieval process

A flowchart of the article retrieval process, annotated with the number of articles that resulted in each stage, is shown in Fig. 1.

#### Identification

When applied to each of the repositories, this search strategy returned 197 articles. Twelve additional articles were identified through reference lists of the 197 articles. The references from four systematic reviews of 85 papers identified in the preliminary search were further reviewed for suitability for inclusion resulting in the addition of eight articles and a total of 217 potential articles. Duplicates (n = 16) were removed. Assessment for eligibility removed 127 articles as they did not meet the preliminary criteria of a game 1) relating to health promotion and 2) targeted at adolescents. This resulted in the assessment of 74 complete articles against the inclusion and exclusion criteria.

#### Included

The outcome of the iterative process led to the selection and review of 21 research articles.

## Results

### Game categories

The 21 selected articles met the eligibility criteria and were categorised as serious health promotion games targeting adolescent health (World Health Organization [WHO], 2016). The articles were reviewed, and games were categorised into three broad areas:

- healthy lifestyle games focusing on nutrition, obesity, physical activity, disease prevention and management (n = 13),
- sexual health games including games focusing on sexually transmitted infections (STIs), sexual relationships and contraception (n = 4), and
- games focusing on substance use including on the topics of smoking, illicit drugs and alcohol (n = 4).

Table 2 provides an overview of each of the papers reviewed. More than half of the games in the review were developed and studied in the USA (Baranowski et al., 2011; Tortolero et al., 2010). Participants’ age ranged from 7 to 29 years with most studies focusing on school-aged adolescents.

**Table 2:** Overview of articles

Author(s), year of publication, Country	Game and category	Study design, population and sample size	Comparator group and activity	Duration of the intervention	Outcomes measured	Key findings that relate to the review question
Baños et al., 2013 (34) Spain	"ETIOBE Mates": Nutrition/diet	RCT. 228 participants aged 10 to 13 years	Yes: received a pamphlet	Two weeks, unlimited play	**Knowledge:** Nutritional Knowledge **Engagement/Enjoyment:** Acceptability–Playability Internet and game-playing habits	All participants increased their nutritional understanding. However, the participants in the group that used *ETIOBE Mates* acquired more knowledge compared with those who accessed the information from the informational pamphlet
Baranowski et al., 2011 (22) United States	"Escape from Diab” (Diab) and “Nanoswarm: Invasion from Inner Space” (Nano) video games on children’s diet, physical activity, and adiposity	RCT. 133 participants aged 10 to 12 years	Yes: played diet and physical activity knowledge-based games on popular websites	Nine sessions, 40 min duration	**Behaviour:** Servings of fruit, vegetable, and water; minutes of moderate to vigorous physical activity Physical activity using accelerometers; and assessment of height, weight, waist circumference, and triceps skinfold	Children playing these video games increased fruit and vegetable consumption by about 0.67 servings per day (p < 0.018) but not water and moderate-to-vigorous physical activity, or body composition
Cullen et al., 2005 (23) United States	"Squire's Quest!": Nutrition (Fruit and Vegetable Consumption)	RCT. 1578 participants aged 8 to 12 years	Yes: ‘Control conditions’ that are not defined	Ten sessions, duration not recorded	**Behaviour:** Fruit, fruit juice and vegetable (FJV) servings consumed by meal	The difference in means was significantly higher for fruit and 100% fruit juice at snacks (both a school and home meal– environment), and for regular, non-fried vegetables at lunch (a school meal–environment) in intervention vs control groups. No significant differences for other FJV groups or other meals
Damasceno et al., 2017 (46) Brazil	Untitled game: Illicit drugs	One sample, qualitative. 69 participants aged 13 (mean)	No	One session, 20 min duration	**Engagement/Enjoyment:** Questionnaire to evaluate the use of the game as an educational resource. Game metrics: how many stages or phases the player passed and the total possible stages of play; the proportion of 'good' and 'bad' responses	Difficult to interpret; overall there were reports of ‘bad’ and ‘good’ responses which are assumed to be ‘incorrect and correct’ relating to correctly answering questions in the game, although the proportions aren’t clear.There was a higher volume of ‘negative’ repsonses than ‘positive’
Fuchslocher et al., 2011 (36) Germany	"Balance": developed to optimize the self-management of teenagers with diabetes mellitus type-I	Two sample, pre-post. 20 participants aged 11 to 16 years	Yes: Played ‘Implicit’ vs ‘Explicit’ version of the same game	One session, 15 min duration	**Engagement/Enjoyment:** The two versions were compared with focus on 'enjoyment' **Attitudes:** Self-efficacy and locus of control with regard to the diabetes self-care were also measured on a 6-point rating scale	The explicit game version was better evaluated than the implicit version with regard to game enjoyment. Participants-perceived similarity to game character, diabetes related self-efficacy, and internal locus of control was higher in the explicit game version than in the implicit version
Guana et al., 2014 (42) Canada	"UnderControl": Reproductive health	One sample, qualitative. 11 participants aged 13 to 20 years	No	One session, duration not recorded	**Engagement/Enjoyment:** Questionnaire measuring difficulty and playability, with qualitative feedback	Wide-ranging results regarding percevied difficultly. The game was overall well-received. The interface was ranked positively. There were suggestions from players about making the game more instructive/intuitive to use
Gupta et al., 2015 (37) India	"SheHealthy:” Polycystic Ovary Syndome	One sample, qualitative. 15 participants (12 adolescent girls, 2 gynaecologists and 1 health activist member)	No	One session, duration not recorded	**Engagement/Enjoyment:** Informal evaluation in the form of a focus group	Participants reported they were ‘satisfied’ by the interface, suggestions for improvement included more rewards and a collaborative approach to increase interest and engagement
Haruna et al., 2019 (43) Tanzania	“My Future Begins Today”: Sexual health education	Quasi-experimental 3-arm RCT. 348 participants aged 11 to 15 years	Yes: received traditional instructional approach. Two experimental arms—game-based learning and gamification	One session, duration not recorded	**Knowledge:** Students’ knowledge measured before and after the intervention/control session	Equal level of sexual health literacy scores (baseline) among the three groups (no significant differences detected for pre-tests). Participants in the gamified learning platforms demonstrated higher average scores on their post-tests than controls (F(2, 345) = 210.43, p < 0.001).Groups playing the games commented positively on the effectiveness of their instructional approach than those in the control group
Kato et al., 2008 (24) United States, Canada, Australia	"Re-Mission": Cancer medication adherence	RCT. 375 participants aged 13 to 29 years	Yes: played commercial game on an unrelated topic	Player guided; mean play time 10.7 h for intervention group and 7.7 h for control (p = 0.042)	**Behaviour:** Adherence to prescribed cancer treatment **Knowledge & Attitudes:** Self-efficacy to manage cancer, knowledge about cancer, health locus of control, stress, and quality of life	Enhances adherence to prescribed oral medication regimens in AYA with cancer (significantly higher chemotherapy metabolite levels [6MMP] in intevention vs control, p = 0.002). Increases in self-efficacy (p = 0.011) and cancer-related knowledge (p = 0.035) among intervention group
Klisch et al., 2011 (25) United States	"Uncommon Scents": Inhalants / Toxic Chemicals	One sample, pre-post. 444 participants aged 11 -13 (middle school students)	No	Five sessions, 45 min duration	**Knowledge:** Content knowledge scale; 30 items that reflected information accessible to the player within the game **Engagement/Enjoyment & Beliefs/Attitudes:** Three scales for satisfaction, game usability, and attitudes toward inhalants	The group mean for content knowledge test scores increased from 13.60 (SD 4.05; 45.3% correct) before game play to 17.63 (SD 6.53; 58.8% correct) after playing the game (p < 0.1). Students’ attitudes toward inhalants changed toward negative (pre-test mean 1.79, SD 0.60; post-test mean 1.65, SD 0.625) (p < 0.1). Group means were 3.75 (SD 0.85) for satisfaction with the game and 3.60 (SD 0.71) for game usability
Lee & Lau, 2018 (44) Hong Kong	“The Drug Detective”: Illicit drugs	RCT. 180 participants aged 9 to 12 years	Yes: control group receives no alternative	One session, 30 min duration	**Knowledge & Attitidues:** Knowledge about drugs, attitudes towards drugs and perceived severity [of] drug abuse	Preliminary findings only. The intervention group had increased knowledge post-test (µ = 4.34, SD = 0.61) when comparing to the pretest (µ = 2.38, SD = 0.56). This group also had increase post-test scored for attitudes (µ = 4.07, SD = 0.55) when compared to pre-test (µ = 3.31, SD = 0.62). There were minor differences for perceived severity of drug abuse. Data for the control group were not presented
Lyles et al., 2017 (26) United States	"Monitor Your Avatar": Body image/obesity	One sample, qualitative. 42 participants aged 15 to 18 years	No	One session, duration not recorded	**Engagement/Enjoyment:** Qualitative feedback on the avatars produced by the game & software usability survey	Players reportedly had positive reactions to the avatar app and being able to view avatars that represented them. All but one participant (41/42, 98%) indicated some level of comfort viewing the avatars, and reported intention to use the app in the future to see how their bodies change over time
Majumdar et al., 2013 (27) United States	"Creature 101": Nutrition/diet/obesity/physical activity	Pre-post matched pair intervention-control study. 590 participants aged 11 to 13 years	Yes: played ‘‘Whyville’’ online game on an unrelated topic(s)	Seven sessions,30 min duration	**Behaviour:** A self-reported, validated, online instrument that measured frequency and amount of targeted behaviors (consumption of sweetened beverage and processed snacks, fruit and vegetable intake, water intake, physical activity)	Intervention students reported significant decreases in frequency (p = 0.011)and amount(p = 0.007) of consumption of sweetened beverages and frequency (p < 0.000) processed snacks compared with the controls. No changes were observed for the other behaviors
McPherson et al., 2006 (39) United Kingdom	"The Asthma Files": Athsma management	RCT. 101 participants aged 7 to 14 years	Yes: asthma information booklet alone	Player guided; 1 or more sessions, total ~ 90 min duration	**Knowledge:** 2-part Asthma Knowledge Assessment (AKA) inventory (developed for this study) **Beliefs/Attitudes:** The Children’s Asthma Locus of Control (CALOC) was used to measure asthma locus of control **Behaviour:** Lung function, use of oral steroids, and school absence	At the 1-month follow-up, children in the intervention group had improved knowledge compared with the control group (p = 0.001), and a more internal locus of control (p = 0.007). There were no differences in objective lung-function measures, hospitalizations, or oral steroid use. The study participants evaluated the intervention ‘postively’ At the 6-month follow-up,, significantly fewer children in the intervention group had required oral steroids (p = 0.026) and had had time off school for asthma (p = 0.034) in the previous 6 months. The difference did not reach statistical significance in the intention-to-treat analysis for both steroid use and school absence
Molnar & Kostkova, 2018 (40) United Kingdom	"MicrobQuest!": Hygiene/infectious disease prevention	One sample, pre-post. 19 participants aged 9 to 12 years	No	One session, 30 min duration	**Knowledge:** 16 statements assessing the students' knowledge acquired during game-play	The results showed that for **one** of the learning objectives, Q8—Bacteria, viruses and fungi come in different shapes and sizes, the difference was statistically significant (p = 0.04). For the other learning objectives, the difference between the pre and post test was not statistically significant
Pentz et al., 2019 (29) United States	PlayForward: smokeSCREEN: Smoking prevention	One sample, pre-post. 80 participants aged 11 to 14 years	No	Four sessions, 60 min duration	**Knoweldge & Beliefs/Attitudes:** Survey with questions adapted from national surveys (Global Youth Tobacco Survey,2002; National Youth Tobacco Survey,2014) on: knowledge • risk perceptions • personal beliefs about use • intentions about cigarettes, e-cigarettes, and other tobacco use	Reported change in knowledge of e-cigarettes and other tobacco products (p’s < . 001), risk perceptions of cigarettes and e-cigarettes (p < .01 and p < .001, respectively), and beliefs about e-cigarettes and other tobacco products (p’s < .05), but not intentions Older adolescents reported greater e-cigarette knowledge and risk perceptions (p’s < .05), and females reported greater risk perception of cigarettes (p < .05). Beliefs mediated the relationship between knowledge and intentions to use e-cigarettes (indirect effect p < .05)
Pernencar et al., 2018 (35) Portugal	"TeenPower": helping overweight teenagers to address lifestyle surveillance, selfmonitoring and social support through the use of a digital platform	One sample, qualitative. 5 participants aged 14 to 17 years	No	One session, duration not recorded	**Engagement/Enjoyment:** How many users completed pre-defined tasks successfully, qualitative feedback on suggestions to improve	Herterogenous reports of difficulties in using the game. Most suggestions were based on contributions to improve the game itself, not on the existing relationship of doing healthy activities when they are having fun
Schinke et al., 2005 (30) United States	"Thinking Not Drinking: A SODAS City Adventure": Reducing alcohol consumption	RCT. 489 participants aged 9 to 13 + years	Yes: 3-arms. Control Not intervention) vs, CD-ROM intervention vs parent-enhanced CD-ROM intervention	Ten sessions, 45 min duration	**Knowledge & Attitudes/Beliefs:** Assertion skills, including self-efficacy, problem solving, educational attainment, peer interactions, and family rules related to alcohol and substance use	Participant scores at pre-test did not differ significantly (Intervention = 4.20, Control = 4.24) when asked to rate their perceived harm of alcohol. At post-test, intervention participants scored higher with a mean of 4.41, whereas control participants scored 4.21, t(485) = 1.94, p < .053 (two-tailed)
Sharma et al., 2015 (31) United States	"Quest to Lava Mountain": Nutrition/diet/obesity/physical activity	RCT. 94 participants aged 9 to 11 years	Yes: control schools had ‘usual programs’	Recommended game exposure duration was 90 min/wk for 6 weeks	**Behaviour:** Dietary intake (self-report) Physical activity, and psychosocial factors **Engagement/Enjoyment:** Process data on game usability and back-end server data on game exposure and progress achieved	Children in the intervention group reported decreased sugar consumption (p < 0.021) and higher nutrition/physical activity attitudes (p < 0.041) pre- to post-intervention. There were no significant effects of the game on physical activity between the two groups. Post-hoc analysis (intervention group only) showed that higher game exposure and gaming progress was associated with increased frequency of physical activity (p < 0.05)
Shegog et al., 2007 (32) United States	"It's Your Game: Keep It Real": Delaying sexual behaviour	One-sample, pre-post. 14 participants aged 12 to 14 years	No	Four sessions, 35 min duration	**Engagement/Enjoyment:** Feedback questionnaites, qualitative	Student attitudes toward the use of computers in education increased 7^th^ and 8^th^ grade samples. Ratings of the importance of the program content in each lesson increased significantly for all content domains (p < 0.05). Ratings of self-efficacy for enacting behaviors in these domains also improved. The game received high ratings for Usability, Ease of use, Credibility, Understandability and Acceptability, measured by proportion agreement with statements measuring these outcomes
Tortolero et al., 2010 (33) United States	"It's Your Game: Keep It Real": Delaying sexual behaviour	RCT. 907 participants aged 13 years	Yes: ‘Control conditions’ that are not defined	Twelve sessions, 45 min duration	**Behaviour:** Delayed sexual initiation (self-report) at the 9th grade follow-up for those students who reported no lifetime sexual activity at baseline Secondary outcome measures • delayed initiation of specific types of sex • delayed sexual initiation by gender and racial/ethnicity, and • 3) reduced risk behavior at the 9th-grade follow-up for those who reported being sexually active in the last three months	Students in the control condition had an ARR 1.76 of initiating oral sex and am ARR 2.67 of initiating anal sex by 9th grade than those in the intervention group (p < 0.05) At the time of the 8th-grade post-intervention survey, students in the intervention condition had more positive beliefs and knowledge across a range of secondary outcomes including knoweldge, beliefs and attitudes

## Research question 1: What educational games could be identified in the literature that are used for health promotion in adolescents?

Of the serious games identified as ‘healthy lifestyle’ games (n = 13), the majority (n = 9) focused on improving nutrition knowledge and increasing physical activity to prevent or combat obesity (Baños, et al., 2013; Baranowski et al., 2011; Cullen et al., 2005; Majumdar et al., 2013; Pernencar et al., 2018; Sharma et al., 2015). Two games were focused on diet and physical activity, specifically addressing the health concerns of type 2 diabetes (Fuchslocher et al., 2011) and polycystic ovarian syndrome (PCOS) (Gupta et al., 2015; Thompson et al., 2010). Other topics under this broad umbrella term included asthma health literacy and management (McPherson et al., 2006), cancer medication adherence (Kato et al., 2008) and communicable diseases (i.e., not specifically STIs), including concepts of immunisation, hygiene, and infection prevention (Molnar & Kostkova, 2018).

Four ‘sexual health’ serious games were identified (Shegog et al., 2007; Arnab et al., 2013; Guana et al., 2014; Hussein et al., 2019). All included information on STI prevention. One game also focussed on delaying initiation of sexual relationships to reduce the likelihood of early pregnancy (Shegog et al., 2007) whereas another considered sexual consent and coercion (Arnab et al., 2013). Four games related to ‘substance use’ were identified, including illicit drugs (Lee & Lau, 2018), inhalants (Klisch et al., 2012), alcohol (Klisch et al., 2012; Schinke et al., 2005) and tobacco (Pentz et al., 2019).

Target groups of minority or disadvantaged adolescent populations included black and Hispanic youth in lower socioeconomic status USA neighbourhoods (Baranowski et al., 2011; Majumdar et al., 2013; Shegog et al., 2007) ‘high risk youth’, that is those living in communities with high drug use and high poverty neighbourhoods (Schinke et al., 2005) and schools with most students on Government subsidised or free school meals (Sharma et al., 2015). Two studies were carried out in developing countries of India: Hussein et al. (2019) reported on a sexual health promotion game with adolescents in Tanzania while Gupta et al.(2015) presented their game on polycystic ovary syndrome that targeted adolescent girls from low-income backgrounds.

Games incorporated elements for learning including; knowledge games with problem solving, decision-making scenarios and goal setting (Baranowski et al., 2011; Gupta et al., 2015; Hussein et al., 2019; Klisch et al., 2012; Majumdar et al., 2013; Pentz et al., 2019; Shegog et al., 2007) platform games (Baños et al., 2013; Fuchslocher et al., 2011), mission or action games with detective/ secret agent themes (Farrell et al., 2011; Guana et al., 2014; Kato et al., 2008; Lee & Lau, 2018; McPherson et al., 2006; Molnar & Kostkova, 2018; Sharma et al., 2015) and a gameshow model (Arnab et al., 2013). The use of ‘avatars’ was common in these games, with mobile apps favoured due to smartphone accessibility.

## Research Question 2: What *methods* were used to evaluate participant engagement and serious games efficacy?

### Engagement and participant satisfaction

Game efficacy is influenced by participant engagement. Engagement in turn relies in part on participant enjoyment and satisfaction. Engagement levels were evaluated in most studies, although heterogenous methods were employed. Arnab et al. (2013) used the term ‘engaged’ to describe student level of discussion in the game, the rate of competitiveness in the game, and participants interest in responding correctly to questions. Alternatively, Hussein et al. (2019) measured participant engagement by asking questions around “consistency, active participation, confidence, fun, excitement, individual attention, clarity of learning, meaningful work, rigorous thinking, and performance orientation” (p14) focusing on enjoyment, learning outcome and quality.

Sharma et al. (2015) asked participants if they would recommend the game to friends and if they would want to play the game again. Pentz et al. (2019) measured participant satisfaction during game play using a 10-point Likert questions such as ‘I feel connected to my character in the game’. Another, and more objective approach to measure player engagement focussed on retention and completion data, with the number of levels of a game completed an indicator of player engagement (Damasceno et al., 2017; Majumdar et al., 2013).

### Efficacy

Four studies did not evaluate game efficacy as an educational tool but presented data limited to game play enjoyment (DeSmet et al., 2014; Guana et al., 2014; Lyles et al., 2017; Pernencar et al., 2018). Seventeen games reported a quantitative evaluation of the efficacy of the game via knowledge, attitudes and/or behaviours. Ten adopted a randomised controlled trial (RCT) design (Ammerlaan et al., 2011; Arnab et al., 2013; Baños et al., 2013; Baranowski et al., 2011; Farrell et al., 2011; Hussein et al., 2019; Kato et al., 2008; McPherson et al., 2006; Schinke et al., 2005; Sharma et al., 2015), six a pre-post design (Klisch et al., 2012; Majumdar et al., 2013; Molnar & Kostkova, 2018; Pentz et al., 2019; Shegog et al., 2007) and one used a between-subjects approach (Fuchslocher et al., 2011). Gupta et al. (2015) employed qualitative methods only, using focus groups to collect data on game efficacy. As with game play engagement, differences were noted in the parameters used to measure efficacy across the constructs. Broadly, efficacy was measured as changes in knowledge, attitude/perceptions and/or behaviour.

### Knowledge

Pre-post intervention knowledge comparison was the most common efficacy measure. The sexual health promotion game evaluation conducted by Hussein et al. (2019) was limited to knowledge acquisition, measured as sexual health literacy through the constructs of motivation, attitude, knowledge, and engagement using a previously evaluated framework with a Likert Scale tool. *PlayForward: smokeSCREEN*, a game designed to prevent tobacco use, applied questions adapted from national tobacco organisation surveys to test knowledge acquisition (Pentz et al., 2019). Baῆos et al. (2013) who limited their efficacy evaluation to nutrition knowledge acquisition in *the ETIOBE mates*, also used a validated tool, which was a modified version of a Nutritional Knowledge Questionnaire (Parmenter & Wardle, 1999), which they adapted for children. The validity of this adapted version was not discussed.

Other researchers developed assessment tools such as Molnar and Kostkova (2018) and Farrell et al. (2011) who assessed the knowledge of personal hygiene in the *e-bug junior* game Alternatively, Klisch et al. (2012) who measured student knowledge and attitude towards asthma inhaler use in *Uncommon scents* did not describe the tool used to measure either criterion.

### Attitudes/perceptions

Attitudes are a pre-cursor to behavioural change (Ajzen & Fishbein, 1980), and therefore an important outcome to consider in any behaviour change focussed intervention. Attitude measures varied among the studies. Lee and Lau (2018) measured knowledge and attitude towards illicit drugs, which included perceived risk of drug use, in their *Drug Detective* game, using self-designed questionnaires for both constructs. In contrast, Klisch et al. (2012) measured attitude change via a validated scale as did McPherson et al. (2006) using a validated children’s asthma locus of control scale.

Self-efficacy (confidence in enacting personal behaviour change) was often used to measure attitude change. Fuscholcher et al. (2011) revised an established scale for blood sugar management self-efficacy in diabetic patients, whereas Schinke et al. (2005) measured changes in participant assertiveness regarding drug use behaviour. Others developed their own self-efficacy measuring instruments using ‘semantic scales’ to ascertain illicit drug consumption behaviours (Shegog et al., 2007) or diet and activity (Sharma et al., 2015). Kato et al. (2008) similarly designed their own self-efficacy scale in their cancer medication regime compliance study drawing on Bandura’s work to develop this tool (2006). Arnab et al. (2013) limited evaluation of their relationship and sexual education game to self-efficacy using 16 questions from their main game objectives.

### Behavioural intention and behaviour

Behaviour changes were less commonly reported, and measurements relied mostly on self-reported dietary behaviours, including consumption of sweetened beverage, processed food, water and fruit and vegetables, and activity and exercise (Baranowski et al., 2011; Cullen et al., 2005; Majumdar et al., 2013; Sharma et al., 2015) and the objective measure albeit self-reported, Body Mass Index (BMI) (Baranowski et al., 2011; Sharma et al., 2015).

Other objective data used to ascertain game efficacy in terms of behavioural change included blood biomarkers to ascertain cancer medication adherence which complemented self-reported drug adherence, stress, control and quality of life (Kato et al., 2008); lung function assessed by clinicians before and after gameplay, and school absenteeism as a proxy measure for medication adherence (McPherson et al., 2006).

## Research Question 3: What does the evidence show in terms of efficacy of these games in increasing knowledge/skills or changing behaviour/attitudes of adolescents?

Most studies that measured efficacy claimed some success in their findings. Gains in knowledge were reported in gameplay related to sexual health literacy (Hussein et al., 2019), nutrition (Baños et al., 2013) and inhalant risks (Klisch et al., 2012). Some studies, however, reported mixed or even negative findings. Knowledge about hygiene in the *e-bug junior* game showed a statistically significant improvement in just one of the 16 learning objectives post-test gameplay (Molnar & Kostkova, 2018). Sharma et al. (2015) reported a significant reduction in sugar intake in the intervention group of a nutrition and exercise game, but there was no change in fruit and vegetable intake, physical activity behaviour, self-efficacy or nutritional knowledge. Others found increases in fruit and vegetable intake in a RCT with a diet and activity game, but no change in water intake and only moderate physical activity change and body composition improvement (Baranowski et al., 2011). Alternatively, no change occurred in any players of *Creature-101,* in terms of fruit, vegetable or water intake or physical activity (Majumdar et al., 2013).

Despite knowledge being a prerequisite for attitude change and attitude change a prerequisite to behaviour change, this relationship was not always seen with these games. Arnab et al. (2013) for example, found the students playing their *PR:EPARe* Relationship and Sexual Education (RSE) game were found to have statistically higher knowledge of personal risk and consequences regarding sexual consent than the control group. However, no statistically significant change in confidence to recognise, act and stop inappropriate sexual behaviour when compared with the control group. Furthermore ‘knowledge and positive attitudes to saying no’ had decreased in the intervention group after game participation. The authors proposed this result may have been influenced by the complex nature of the concept of coercion which, as a newly introduced idea with most of the pupils, may have reduced their feelings of confidence in the subject. Pentz et al. (2019) found knowledge, perceptions of risk around smoking were significantly different post intervention, but the intent to smoke was not. Schinke et al. (2005) similarly found increased knowledge and perception of the harms of alcohol but no change in intentions to use alcohol.

Sharma et al. (2015) found a significant positive shift in attitude towards healthy foods in their nutrition and activity game *Quest to Lava Mountain* (Lyles et al., 2017). This did not, however, translate to a change in attitude or behaviour in dietary patterns, physical activities or self-efficacy. In contrast, Cullen et al. (2005) found students who completed their *Squire’s Quest* interactive game reported a statistically higher fruit and vegetable intake in lunchtime meals than the control group.

Locus of control, measured by McPherson et al. (2006) in *The Asthma Files*, showed statistically significant increases in the intervention group compared to control group and Shegog et al. (2007) found self-efficacy regarding sexual behaviours of the participants significantly improved. Despite Kato et al. (2008) finding higher adherence to cancer medication when measured objectively (blood biomarkers), there was no significant difference in self-reported drug adherence. This study also reported no significant difference in self-reported stress, control or quality of life, suggesting complex interactions between a player’s personal attributes including health status and concurrent mediations may mediate outcomes. This apparent discrepancy between self-report and objectively measured outcomes highlights potential limitations in the use of self-report measures.

### Influences on serious game efficacy

Analysis of the 21 articles in this review identified several influences on game efficacy—some reported by the researchers. Further influences determined by the authors of this review include game design and research design issues.

Some studies were unable to be adequately assessed due to incomplete data. An example can be seen in the limited data presented in Lee and Lau (2018) drug detective game, with no comparison of data between control group and intervention group. It is therefore difficult to draw conclusions on the efficacy of the game and the effects on knowledge and attitudes. Gupta et al. (2015) also provided no data to support their evaluation findings in *SheHealthy*, either for engagement or knowledge gain. This highlights the need for transparency and rigour in the presentation of results, to ensure conclusions can be supported with empirical data.

### Game design

From the analysis a professional commercial company to design a game was a recognised benefit including enhanced participant engagement and efficacy (Hussein et al., 2019; Schinke et al., 2005; Sharma et al., 2015), and high-quality graphics (Schinke et al., 2005). Other games with poorly defined graphics (Pernencar et al., 2018) or simplistic design (Baños et al., 2013) reported limited participant engagement and enjoyment (Baños et al., 2013; Pernencar et al., 2018). Further a multidiscipline approach to game development improved game authenticity, game quality (Fuchslocher et al., 2011) and acceptability (Arnab et al., 2013).

### Multidisciplinary game design and end user contribution

Some articles described the use of a multidisciplinary approach to game development to improve the relevance and validity of the game design. For example, Hussein et al. (2019) worked with a multidisciplinary team of paediatricians, sexual and reproductive health specialists, computer science specialists and sex education teachers, as well as end users (school children) to ensure the game was ‘reliable and relevant’. Other examples include psychologists who applied positive reinforcement activities Fuchslocher et al. (2011) and Gupta et al. (2015) interviewed female gynaecologist and a social health activist in India to develop a critical and culturally informed polycystic ovary disease awareness app.

## Theoretical framework

Theoretical frameworks were used to improve authentically and learning. Hussein et al. (2019) argued the success of their game was in part related to their use of socio-cultural activity theory which encourages stakeholder collaboration in game design (Hung & Wong, 2000). Fuchslocher et al. (2011) argued the application of social learning theory to game design had influenced the superior outcome of their explicit diabetic health promotion game. In this study, the self-efficacy was greater in diabetic participants who played the ‘explicit’ version of *Balance* (where the character also had diabetes) compared to those who played the implicit version (where diabetes was not mentioned). The researchers argued “the perceived similarity between participants and game character had facilitated vicarious learning processes leading to the reported rise in diabetes management self-efficacy” (p.100).

Many games were based on a mix of these theories, for example Lee and Lau (2018) used the theory of reasoned action, the health belief model, and the theory of planned behaviour to evaluate their game that explored the relationship between participants behaviours, attitudes and intentions regarding illicit drugs. Almost half (n = 10) the articles, however, did not mention any theoretical underpinning to their game design or development (Baranowski et al., 2011; Farrell et al., 2011; Guana et al., 2014; Gupta et al., 2015; Klisch et al., 2012; Lyles et al., 2017; McPherson et al., 2006; Molnar & Kostkova, 2018; Pentz et al., 2019; Pernencar et al., 2018).

### Teaching approaches for complex issues

Two authors argued the subject material complexity may have negatively influenced game efficacy. Arnab et al. (2013) argued the complexity of the topic of coercion made it unsuitable for delivery by game format. Similarly, the lack of change in post-test perception of risk associated with a game about illicit drug taking may have been due to the difficulty of delivering this complex topic though the medium of gaming (Lee & Lau, 2018). Alternatively, the virtual approach in *My Future Begins Today* by Hussein et al. (2019) was viewed positively, with the authors pointing out the confidential individual nature of interaction during gameplay facilitated engagement with this sensitive and sometimes taboo subject of sexual health.

### Ceiling effect

Reaching the ‘ceiling effect’ through repeat and consistent education was considered by some researchers as a potential reason for a lack of post-intervention increase in knowledge such as tobacco-related health issues with Pentz et al. (2019) and hygiene knowledge with Farrell et al. (2011). The ceiling effect was also cited by Fuchslocher et al. (2011) who attributed significant learning of tobacco smoking health dangers, which may have been taught in other classes, as the reason behind the lack of knowledge increase among students who played their game.

### Teacher support

Practical teacher support including co-delivery or classroom management was identified as important to game success (Arnab et al., 2013) whereas others reported a lack of teacher support was related to the limited nutritional knowledge gain between the intervention and control groups (Baños et al., 2013), and knowledge and attitude change post intervention (Klisch et al., 2012).

### Setting

Gameplay when aligned or integrated to relevant curriculum both temporally and contextually lead to greater efficacy. This was found in curriculum areas of science (Klisch et al., 2012) and sexual health (Shegog et al., 2007). When games were either outside the relevant discipline curriculum and class time (Farrell et al., 2011) or related to prior learning gameplay had reportedly little effect on change in knowledge (Farrell et al., 2011).

### Social determinants

Social determinants are an important influence on the efficacy of serious gameplay with children and adolescents, and nutrition being a particular example. Cullen et al. (2005) postulated the lack of efficacy of their nutrition game was in part influenced by the poor food environments high junk food outlets in some pupils’ neighbourhoods. The fact that the children in the intervention group only increased fruit and vegetable intake at lunch time whilst at school is supportive of this argument, others reported other explanatory factors including parents as the gatekeepers of food in the family home (Sharma et al., 2015). Access to gaming devices including mobile phone apps is another issue related to demographic inequality. Access to online resources to promote health literacy in poorer communities, is only possibly if users own a smart phone as was the case in a study in India (Shegog et al., 2007).

Gender also influenced pupil exposure to and engagement with games, indicating the need to consider this social construct when designing and delivering future games. Engagement, according to participant gender, was also interpreted through analysis of demographic data of these articles. Male adolescent participants were at times overrepresented in the samples. Baranowski et al. (2011) and Hussein et al. (2019) reported 56% and 55% of their sample as male 55% as male, respectively. The study sample in Fuchslocher et al. (2011) included 12 males and eight females.

A few reasons for this inequity were proposed by the researchers. Kato et al. (2008) for example proposed the appeal of computer games to males led to an over representation of males in their sample (at 68%), although intervention effects by gender were similar in the outcome measures. Baῆos et al. (2013) reported although playability and acceptability were found to similar between genders in their study, boys found the platform game aimed at reducing obesity, ‘easier’ than girls. The traditional gender difference in academic subjects studied may be a further reason boys participated more than girls in these games. Quest to Lava Mountain developed by Sharma et al. (2015) for example, reported 57% male participation and was set during computer studies classes, which can be male dominated (Zagami et al., 2015). A few exceptions to this trend were noted. Lyles et al. (2017) reported 67% female participation in avatar app game. This pilot study required both parental consent and participant assent, and it is worth noting that given the topic of perception of body shape/image the imbalance may have been influenced by a perception among participants that this is a gender-related issues predominately impacting females, and subsequent participation. Pentz et al. (2019) reported 61% female participation in their tobacco prevention video game; although there was no exploration of this difference, their pre-post single-group study found female gender was significantly associated with greater observed increases in cigarette use risk perception.

### Multiplayer platforms

Several authors emphasised the potential for a multi-player element of a game to improve engagement. Shegog et al. (2007) discussed multiplayer options to increase peer engagement and game uptake. Others suggested the addition of a ‘friends circle’ to create a ‘complete social experience’ (Gupta et al., 2015). For younger children, gameplay with family, may also help parental health literacy and decisions around food choices (Sharma et al., 2015). A lack of empirical evidence in the studies as to the effect of multiplayer options suggests this is something that requires further study. Despite social aspects of gaming being an area of future research, the nature of some health promotion topics such as sexual health and drug and alcohol use may not be suitable for a shared gaming experience. Developers should consider the potential for multi-player aspects to games to both engage and *dis*-engage players, with careful consideration as to the nature of the topic and the likelihood of this influencing player engagement. Some gameplay that lasted between 30 min (Molnar & Kostkova, 2018) to 40 min (Farrell et al., 2011) was sufficient to observe improvements in a small number of learning objectives. Others, however, acknowledge their one-off intervention could not determine the game increased knowledge retention of healthy diet and exercise regimes over time (Baranowski et al., 2011). Longer gameplay around 3 h (Klisch et al., 2012) to 3.5 h in total (Majumdar et al., 2013) also showed limited efficacy (Klisch et al., 2012) or behaviour change in activity and healthy food intake (Majumdar et al., 2013). Lyles et al. (2017) proposed their one-off pilot session limited the external validity of their findings.

Although other interventions lasted several weeks, the researchers in these gameplay studies also cited insufficient game play time as a contributing factor for disappointing efficacy measures. Shegog et al. (2007) in the study of *It’s your game*, which focussed on sexual health was played over eight lessons, however, the convenience sample (n = 14) was too small to assess the efficacy of these repeated sessions on knowledge. Conversely, Pentz et al. (2019) reported results of a larger (n = 80) pre-post study conducted over four weeks and reported significant changes in knowledge of e-cigarettes and other tobacco products, risk perceptions of cigarettes and e-cigarettes, and beliefs about e-cigarettes and other tobacco products.

Sharma et al. (2015) also proposed one reason for the lack of increased uptake of fruit and vegetables in the children who played their *Quest to Lava Mountain* nutrition focussed game may be due in part to the short duration of time the students played the game (90 min a week for six weeks). Baῆos et al. (2013) similarly argue the short time frame of two weeks intervention may have influenced the limited difference in nutritional knowledge gain between their intervention and control group. These mixed results suggest the duration of game play or repeated play alone do not solely contribute to efficacy, however, need to be considered in the bigger picture of the complexity of the topic, participant characteristics, and ability to change knowledge versus behaviour. To this end, developers of serious games need to consider the objective of the game and the ability of the content to affect change during development and when considering the duration of the intervention.

### Interval between game play and measurement of outcomes

The studies reviewed had different intervals between gameplay and measurement of outcomes, with one-off studies tending to assess knowledge and/or attitude changes either immediately or soon after the intervention. It was not uncommon for measurements to be taken immediately after gameplay. Pentz et al. (2019) described a pilot investigating the ‘short-term’ effects of a videogame aimed at reducing tobacco smoking in adolescents on knowledge, risk perceptions, beliefs, and intentions. Shegog et al. (2007) similarly acknowledged the limitations of their pilot study, describing their findings of increased self-efficacy regarding sexual behaviours as ‘short-term’.

McPherson et al. (2006) in contrast, carried out a longitudinal study at baseline, one month and six months post intervention with an interactive computer package for children with asthma. The authors found increased knowledge of asthma self-care in children in the intervention group compared to controls. At six months significantly fewer of the intervention group had time off school due to their asthma and lower requirement for steroid medication.

It is reasonable for studies designed to assess behavioural change to allow a longer interval over which change can be made and sustained. Studies with immediate/short-term follow up, however, do not allow assessment of enduring changes to knowledge and attitudes over a long period of time, and so long-term impacts cannot be assessed. This limitation was noted by researchers (Shegog et al., 2007).

### Attrition

A high incidence of drop out and non-completion of games and game levels is both an indicator of game efficacy and a factor limiting the determination of game efficacy reported as affecting the validity of findings.

Majumdar et al. (2013) reported 36% non-completion of all levels of their games, making these participants ineligible for inclusion in data analysis. Reasons proposed for non-completion included absenteeism and slow game downloading. Given limitations of a short, one-off game session reported by others as a potential impediment to efficacy, this suggests an important obstacle for developers of serious games. On one hand, a single session of short duration might not be sufficient to convey educational messages and allow knowledge retention, but on the other, multi-session gaming with many levels and a longer duration of play might reduce attrition, with players not making sufficient progress in the game to hit all learning objectives. Further research using lesson plans of different durations and repetition, while maintaining the same platform and key messages, are crucial to better understanding the duration of play and number of lessons required to optimise efficacy.

Kato et al. (2008) for example reported an attrition rate of 17% over the three months of game play study. The *e-bug junior* game recorded a 50% drop out before completion of stage one of the game and for those who continued, around 50% dropped out in each successive level without completion (Farrell et al., 2011) and only 3% of participants completed level five. Klisch et al. (2012) in a substance use serious game study, lost 167/610 (27%) of participants because of drop out or poor adherence to game play due to absenteeism who, according to the authors, were the pupils likely to be most in need of the educative learning on drug (inhalants) avoidance provided through the game intervention, suggesting students who are more likely to be absent may be those at most risk. Majumdar et al. (2013) lost 120 potential students when one of the participant schools withdrew from the study, which highlights potential difficulties with attrition with enrolling classes or schools into a study, rather than individuals.

### Social desirability bias

As noted, for games that sought to measure changes in behaviour, self-reported behaviour was commonly used to measure outcomes. While this is the most practical method for collecting these data, it is likely the subjects of many of these games; including drug/alcohol use and sexual behaviours and even diet, may not be accurately reported if the participants feel a sense of shame, or distrust the confidentiality of the data collection.

Livingstone et al. (2004) found children tend to underreport dietary intake, especially those who are overweight. This may provide answers to the contradictory findings reported by Sharma et al. where most overweight participants reported a reduction in dietary sugar, but their physical body measurements did not alter (Sharma et al., 2015). Another nutrition game, *Creature-101*, using self-reported behaviours, discounted social desirability as a potential influencing factor on results because ‘changes were seen in some behaviours and not others’ (Majumdar et al., 2013). While this may be the case, this does not consider that participants may be more willing to be honest about some behaviours over others.

Cullen et al. (2005) also reported significant increases in fruit and vegetable intake using a self-report measure (the Food Intake Recording Software System), however this was only observed during school hours. As the authors note “*That the changes occurred at school lunch and snacks, but not at breakfast or dinner suggests that children might have more control over foods available at these times, and that the targeted intervention messages were received, processed, and implemented*” (p. 150). The fact that students did not report increased intake outside the school environment suggests social desirability did not influence these results, and there was a true chance in behaviour observed in the intervention group.

#### Sample size

Several studies were described as ‘pilots’, often with a low number of participants (Lyles et al., 2017; Molnar & Kostkova, 2018; Pentz et al., 2019; Sharma et al., 2015; Shegog et al., 2007). Pilots generally have small sample sizes not powered to detect significant statistical differences unless the effect size is very large, and many studies did not demonstrate any significant differences between the intervention and control groups (or pre-post-test). The pre-post-test study by Shegog et al. (2007) for example, had a single group convenience sample of 14 students and appropriately suggested caution in interpreting results. Lyles et al.(2017) recruited 10 participants.

The pre-post intervention study of the adapted *e-bug junior* game by Molnar and Kostkova (2018) included 19 participants, which the authors admitted contributed to the lack of statistical significance in their measures of game efficacy. The between-subjects study by Fuchslocher et al. (2011) found although diabetic children who played a game explicitly focussed on diabetes messages reported a higher level of self-efficacy and locus of control over their blood sugar self-care than those who played the more generic game without diabetes specific messages, low sample size of meant no statistical significance could be drawn.

In contrast, with the exception of Sharma et al. (2015) (*n* = 44), every RCT study had over 100 participants, with Schinke et al. (2005) recruiting 489 and Kato et al. (2008) recruiting 375 participants. The largest study was by Cullen et al. (2005) which recruited 1,578 participants. Statistically significant outcomes were noted in many of the RCTS. Sharma et al. (2015) for example reported significant decreases in sugar consumption and higher nutrition/physical activity attitudes and Schinke et al. (2005) reported significant assertiveness and perceived harm scores in their alcohol abuse prevention game post intervention group. These findings support the need for well-designed, rigorous studies with suitable sample sizes and sufficient follow-up to demonstrate efficacy across a range of outcomes.

## Discussion

The health issues foci of these serious games unsurprisingly reflected the issues important to youth public health, including the global youth obesity epidemic ([WHO, 2018), problematic alcohol and other drug use (United Nations Office on Drugs and Crime, 2018), sexually transmitted infections and sexual relationships, including the very topical issue of consent (Australian Institute of Health and Welfare [AIHW], 2020). Designs tended to be platform, mission and problem-solving games. Avatars were also popular and seen to support engagement, reflecting the scope of commercial games attractive to students (Bailey & Blackmore, 2017).

The review clearly highlights how professional designers can influence game efficacy, by meeting the demands of a sophisticated digitally literate target audience. The current and ongoing COVID-19 associated debt crisis affecting higher education (Friga, 2020), however, may limit the realisation of this goal with future serious game designers.

The importance of multidisciplinary approach to designing health promotion games is also evident. For medical issues such as asthma and cancer medication in Kato and McPherson et al.’s games, this supports safety. This does raise questions about the time and logistical effort required to develop such games, plus the need for a coherent and productive team mentality. The importance of involving end-users in game development was highlighted in this review, suggesting contribution from the target demographic is crucial to serious game authenticity and success.

Germov (2009) notes a key objective of health promotion is social justice. This viewpoint was evident in the strong focus of disadvantaged and marginalised target groups of some of the serious games reviewed. These target groups tend to be subpopulations within Organisation for Economic Cooperation and Development nations rather than the population of poorer developing countries. As most of the studies are based in the USA, the ethnicity of these participants tended to be African American and Hispanic. Low-income neighbourhoods are also targeted in several studies where there is known to be a disproportionate level of unsafe sexual behaviour, drug use and obesity (Heronet al., 2015; Noonan, 2020; Peretti-Watel, et al., 2009). Rigorous health promotion games require behaviour change theory but also need an awareness of social determinants impacting on health. This was raised by two research groups (Cullen et al., 2005; Sharma et al., 2015) who identified the importance of family in decisions around nutritious foods and their influence on game efficacy with adolescents. Gupta et al. also raised the issue of poverty and the importance of the app as an accessible alternative to the home computer (Gupta et al., 2015).

There was a stated importance of building on theory for success, with ten articles describing one or more behavioural theories from sociological and psychological disciplines underpinning game design and, in some cases, game evaluation. Hussein et al. and Hung & Wolf for example, explained how activity theory was used to ensure their sexual health game was based on ‘sound theoretical framework’ (Hung & Wong, 2000; Hussein et al., 2019). The usefulness of these theories as predictors of behaviour changes and attitude shift was demonstrated in several evaluations, including Fuchslocher et al. (2011) who provided a clear understanding of how social learning theory could be used to interpret the success of their content-explicit game over their content-implicit game approach. The use of behaviour change theory are highly valuable in behaviour modification including diet (Diep et al., 2014) and social cognitive theory, for example, was a popular choice to ensure knowledge acquisition, agency, and behaviour change (Bandura, 2006). In considering the importance of ‘theory’ cited by some researchers, it is somewhat concerning most of the articles reviewed did not mention any theoretical guide or theoretical basis to their game design.

The importance of the social aspect of gaming, in the form of multi-play between friends, peers or family can be explained by social learning theory and was highlighted in three review articles, with recommendations for multiplayer learning coming from both researchers and players. This approach, if extended to family, may also overcome some of the lack of autonomy of adolescents in behaviour change through increased health literacy of family decision makers.

Game evaluation also highlighted how teacher support and the context of the gaming sessions could influence efficacy, with researchers arguing the presence of a teacher and gameplay within the subject curriculum was most effective. There also seems to be an argument for avoiding complex issues in serious games content, and for an understanding of prior learning when designing these games.

Time engaged in a game, both the time of the individual session and the number of sessions in a series, were factors also identified as important, with short and one-off interventions perceived as less effective across knowledge acquisition, behaviour and attitude change.

The heterogeneity of both the study designs and game designs, however, limits the ability to generalise the findings of this review. The measures used engagement evaluation were somewhat arbitrary in many cases, developed by the authors with no reference to a standardised framework. Engagement was ‘measured’ using a wide range of criteria including fun, willingness to recommend to others and to play again, active participation, competitiveness, and connection with avatar or character. The lack of a standard definition or framework for game engagement could be problematic for researchers wishing to compare the ‘engage-ability’ of serious games.

Instruments to measure knowledge, attitude and behaviour also varied, some applying and adapting validated tools, others using tools developed by the researchers. The level of heterogeneity in serious game design and evaluation is likely to have a significant impact on the range of their success. Efficacy within and across games was highly mixed in these studies and it was unusual to find a wholly positive evaluation.

The high drop-out rates and non-completion of games and game stages suggests issues with engagement. The resultant loss of data from these studies threatens validity of statistical findings. Small sample sizes in many of the pre- and post-test interventions limited the ability to detect statistical differences between intervention and control groups. Further, issues of attrition and study designs that either used small convenience samples, or had no control group, limit the ability to draw conclusion about serious games efficacy against stated endpoints.

Finally, many of the serious games showed promise in the short term, but could not demonstrate lasting change, in knowledge, attitude or behaviour. This suggests evaluation of serious game efficacy requires a longitudinal study design. This review demonstrates the diverse and disparate way serious game efficacy is evaluated. Approaches can be quantitative and qualitative, and the constructs of engagement, knowledge, attitude and behaviour measured against a series of different constructs.

## Conclusion, implications for research and practice

This systematic scoping review identified a range of serious games targeted at several health promotion topics concerning adolescent health. The games could be broadly grouped into three core topics, with the largest number of games found to promote a ‘healthy lifestyle’ either through improved nutrition knowledge or increased physical activity.

Although the somewhat heterogenous nature of the interpreted research limits the generalisability of our findings, it appears there is a reasonable body of evidence to suggest professionally designed serious games with theoretically underpinning, multiple stakeholders and contextualised to curriculum, may be effective in increasing knowledge and influencing attitudes. Limited evidence is available, however, about the impacts of these games on behaviour, particularly in the longer term.

The limited and inconsistent evidence of serious game efficacy in delivering educational content, may constrain use among educators, particularly teachers. This review highlights the need for formal game evaluation. Rigorous methods will allow for meta-analysis and thus, lead to future guidelines for the development of serious games.

## Data Availability

The datasets generated during and/or analysed during the current study are available from the corresponding author on reasonable request.
